# Anti Mullerian Hormone: Ovarian response indicator in young patients receiving Long GnRH Agonist Protocol for Ovarian Stimulation

**DOI:** 10.12669/pjms.324.10267

**Published:** 2016

**Authors:** Zehra Jamil, Syeda Sadia Fatima, Rehana Rehman, Faiza Alam, Sara Arif

**Affiliations:** 1 Zehra Jamil, Department of Biological & Biomedical Sciences, Aga Khan University, Karachi, Pakistan; 2 Syeda Sadia Fatima, Department of Biological & Biomedical Sciences, Aga Khan University, Karachi, Pakistan; 3 Rehana Rehman, Department of Biological & Biomedical Sciences, Aga Khan University, Karachi, Pakistan; 4 Faiza Alam, Department of Biological & Biomedical Sciences, Aga Khan University, Karachi, Pakistan; 5 Sara Arif, Intern, Civil Hospital, Karachi, Pakistan

**Keywords:** Anti Mullerian Hormone, Assisted Reproductive Technology, Ovarian stimulation, Ovarian reserve

## Abstract

**Objective:**

Anti Mullerian hormone (AMH) is gaining place as ovarian marker, chiefly in infertility assistance. We explored its correlation with oocytes retrieval after long GnRH agonist protocol for stimulation, in younger and older infertile population.

**Methods:**

This retrospective analysis compiled data of 166 females, receiving ICSI treatment from June 2014 to March 2015. Serum FSH, LH, Estadiol, AMH and antral follicle count were assessed. Outcomes were measured as good (5 to 19 oocytes) and bad responders.

**Results:**

Higher discriminatory power of AMH (AUROC; 0.771; p < 0.05) was seen in comparison to FSH (0.692; p < 0.05) and AFC (0.690; p < 0.01). AMH reported strongest association with oocyte retrieved (odds ratio of 15.06). Subgroup analysis reported 68.6 % risk of bad response with AMH levels of less than 1.37ng/ml. This association was observed more significant in young infertile patients <35 year of age (r=0.245; p=0.012) versus older population >35 year (r=0.169; p>0.05).

**Conclusion:**

Our study reaffirms that serum AMH correlates well with oocytes retrieved, particularly in females younger than 35 years. We suggest incorporation of AMH in baseline assessment of infertile females, who are falsely advised to postpone interventions based on their age and normal FSH levels.

## INTRODUCTION

Fertility of females resides in the pool of primordial follicles, they are born with. Amongst these, 30 to 50 follicles are recruited with each menstrual cycle leading to decline in fertility after the age of 30 years.^1^ In cases of infertility, evaluation of ovarian reserve (OR) is essential to optimize protocol for assisted reproductive technique (ART) and prediction of response to counsel the couple.^2^

The likelihood of successful ovarian response is usually assessed on age; most dependable variable affecting results of treatment. However, even within comparable ages, wide variability has been reported,^3^ concluding age as a weak reflector of ovarian pool. Thus other markers such as Follicle stimulating hormone (FSH), luteinizing hormone (LH), Inhibin, Estradiol (E_2_), ovarian volumes and antral follicle count (AFC) have been deployed to predict the response.^4^

A rise in FSH levels is most widely recognized hallmark of reduction in the OR.^5^ It may not be the best option as it suffers inter and intra cyclic fluctuations.^6^ FSH production is further deranged in patients receiving oral contraceptive pills, in polycystic ovarian syndrome (PCOS) and pituitary tumors.^7^ Similarly, AFC is assessed for estimation of dosage and in predicting response to stimulation but operators’ variability, mechanical consistency and previous history of ovarian surgery are its main limitations.

Anti Mullerian hormone (AMH) is recently considered as a unique OR marker, solely secreted by granulosa cells and accurately reflects primordial follicle.^8^ It is a glycoprotein dimer belonging to TGF-β family and has potential role in the maintenance of OR.^9^,^10^ Drop in serum AMH reliably indicates decline in ovarian function. Hence, it is now being used to assess ovarian injury induced by chemotherapy, radiation therapy or ovarian surgery.^11^ It correlates well with the number of oocytes retrieved after stimulation and reflects excessive follicular growth in women with ovarian hyper stimulation syndrome (OHSS) and PCOS.^12^,^13^ These oocytes retrieved after stimulation, reflects availability of embryos for transfer and hence an optimum response to Long GnRH agonist protocol is said to achieve by retrieval of at least five oocytes.^14^

Therefore, this study aimed to assess reliability of various markers (like FSH, AFC and AMH) in predicting response to intra-cytoplasmic sperm injection (ICSI). Our results propose that regarding number of oocytes retrieved, AMH is a better predictor in comparison to age, BMI, AFC and FSH, particularly in younger infertile population (20 to 35 years).

## METHODS

In this retrospective cross-sectional analysis, data of 166 infertile females was collected from Australian Concept Infertility Medical Centre (ACMIC). Females booked for the first ICSI treatment from June 2014 to March 2015, aged 20 to 42 year, with regular menstrual cycles, no endocrine disorders or prior ovarian surgery, were included while PCOS were excluded from study. Ethical clearance was obtained from ACMIC. Written consent was waived by Institutional review board as retrospective design could not alter the clinical decision made during ICSI treatment. Furthermore, patients’ records were anonymized prior to analysis to maintain confidentiality. Serum samples were collected on days 2–3 of menstrual cycles one month prior to ICSI treatment. Supernatant fluid was used for the assays maintained at temperature from 2-8 ºC.

AMH was measured using AMH Gen 11 Elisa reagent kit (Beckman coulter, ref a79765). FSH and LH were measured using Elecsys reagent kit following the manufacturer protocol. AFC assessment was carried out onday 3, using an Aloka SSD-1000 (Japan) with a 5 MHz transvaginal probe. Follicles measuring <10 mm in diameter were counted in both ovaries to determine the cohort with an inter observer CV <5%.

1 mg of subcutaneous Buserelin Acetate (Suprefact) was initiated on day 21 to achieve adequate ovarian suppression, followed by recombinant FSH (Gonal-f) or hMG (Menogon). hCG (Ovitrelle, 250 μg, Merck Serono) was administered on an adequate E_2_ response and two or more follicles measuring ≥18 mm. Oocytes were retrieved trans-vaginally and sperms were injected. Embryos were transferred into uterus after 72 hours of insemination. Luteal phase support was given by Pregnyl 1500 IU. Clinical pregnancy was confirmed by sonographic evidence of an intrauterine gestational sac.

Main outcome was measured as good responders having at least 5 to 19 oocytes retrieved while bad responders had either less than 5 or more than 19 oocytes retrieved. Positive pregnancies were not considered as the outcome due to limited size of sample and inclusion of cases with male infertility.

Data was analyzed by SPSS version 19 and comparison of variables was done by Mann Whitney U test. Logistic regression determined the predictive value for ovarian response while AUROC analysis was used to see the predictive accuracy of FSH and AMH. Spearman bivariate correlation was applied between AMH and FSH. P value of <0.05 was considered significant.

## RESULTS

The mean BMI, FSH and AMH levels of one hundred and sixty-six infertile females recruited in this study is summarized in Table-I. While grouping them according to oocytes retrieved, both AMH and AFC were significantly low in bad responders (p < 0.05) whereas FSH was raised (p <0.05).

**Table-I:** Descriptive statistics of whole cohort and according to responder category.

Variables	Whole Study Population	Good Responder (5-19 oocyte count)	Bad Responder (<5, >19 oocytes)

	n =166	n =90	n = 76

		Mean ± SD	
Age (year)	33.6 ± 6.03	31.7 ± 5.4	35.6 ± 6.0
BMI (kg/m^2^)	29.3 ± 5.41	28.70 ± 5.4	29.8± 5.3
FSH (IU/L)	8.5 ± 4.9	6.9 ± 3.2	10.2 ± 5.7*
LH (IU/L)	6.9 ± 1.057	6.5 ± 1.7	7.4 ± 1.4
AMH (ng/ml)	1.6 ± 1.3	2.3 ± 0.7	1.4 ± 0.5*
AFC	9.3 ± 4.3	10.6 ± 5.1	7.9 ± 2.6*
Estradiol (pg/ml)	46.95 ± 6.3	48.0 ± 6.78	45.0 ± 7.3

Data expressed as Mean ± S.D. Mann Whitney U test was used to compare the difference between groups

Table-II compares the groups according to AMH cut off, FSH and age. One hundred fifty two females reported low AMH and out of these, only 47.5% responded well while in higher AMH category, 85.7% females had a good response. In these groups, significant difference in AMH was observed (p<0.01) whereas LH, FSH and AFC had insignificant difference. On FSH stratification, significant difference in the levels of LH, AMH and AFC (p <0.05) was observed. Good response was reported by 62.2% of normal while 30.0% of raised FSH group. In age segregated groups, insignificant variance was seen for AMH, LH and FSH.

**Table-II:** Biophysical and Biochemical Variables on the basis of AMH, FSH and Age Category.

	Age		AMH		FSH	

	35-42 year n = 60	20-35 year n =106	Normal >1.37ng/ml n =14	Low <1.37ng/ml n = 152	Normal < 11 IU/L n =106	High >11 IU/L n = 60

Mean ± SD						
Age (year)	40.2 ± 3.5	29.9±3.4*	33 ± 8.9	33.6 ± 5.7	32.3 ±5.7	36 ± 6.0
BMI (kg/m^2^)	30.1 ± 5.2	28.8±5.5	33.4 ± 3.3	28.9 ± 5.4*	28.9 ± 5.1	30 ± 5.6
FSH (IU/L)	9.1 ± 5.3	8.1 ± 4.6	5.9 ± 3.3	8.8 ± 4.5	6.1± 1.9	13 ± 5.5*
LH (IU/L)	7.3 ± 2.5	6.8 ± 1.1	4.3 ± 2.5	7.2 ± 1.5	4.5 ± 2.4	11.3 ± 4.4*
AMH (ng/ml)	1.5 ± 0.8	2.0 ± 0.3*	2.8 ± 1.1	0.7 ± 0.3*	2.2 ± 0.4	1.2 ± 0.5*
AFC	8.4 ± 2.9	9.8 ± 4.8	8.0 ± 1.9	9.4 ± 4.4	8.6 ± 4.6	9.7 ± 4.0*
Good Responder%	36.6	58.5	85.7	47.4	62.2	30.0
Bad Responder %	63.3	41.5	14.2	52.6	37.7	70.0

Data expressed as Mean ± S.D. and frequency and percentage wherever applicable. Mann Whitney U test was used to compare the difference between groups.

* p<0.05 considered significant.

Next, on binary logistic regression, unadjusted model reported that females with low AMH were 6.66 times more likely to have a poor response than raised FSH (3.66 times). After adjusting for age and BMI, AMH gave even stronger positive significant association with the responder group [OR 15.06 (2.83- 80.01)] versus FSH [OR 4.12 (1.12-9.86)] (Table-III).

**Table-III:** Logistic Regression Analysis for FSH, AMH and AFC.

Variables	Unadjusted			Adjusted for Age and BMI		
	
	OR	95% C.I		OR	95% C.I	

		Lower	Upper		Lower	Upper
FSH	3.66*	1.85	7.24	4.12*	1.72	9.86
AMH	6.66*	1.44	30.80	15.06*	2.83	80.01
AFC	0.79*	0.70	0.89	0.81*	0.72	0.90

**Fig.1:**
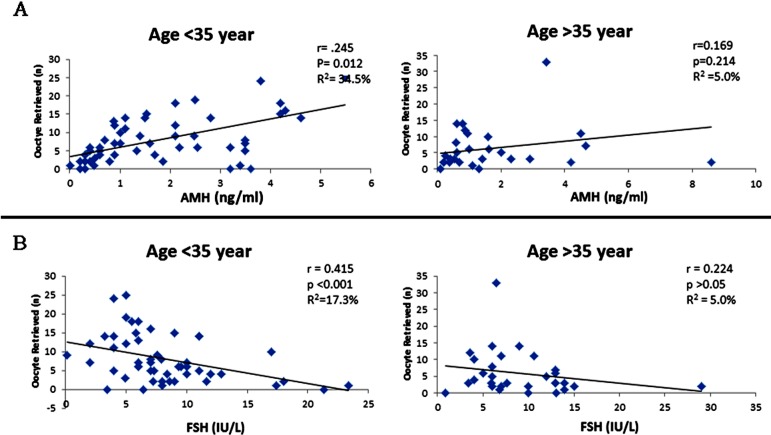
Correlation of Serum AMH (A) and Serum FSH (B) with the number of oocyte retrieved after stimulation. Patients grouped according to ages between 20 to 35 year and 35 to 42 year.

Fig.1 presents correlation of AMH and FSH with oocytes retrieved in various age groups. AMH depicted stronger positive association in patients <35 year of age (r=0.245; p=0.012) versus patients >35 year (r=0.169; p>0.05). FSH depicted negative correlation that was likewise higher in patients <35 year of age (r=-0.415; p<0.001). In addition, significant negative correlation with bad responders was observed between the oocyte retrieval and AMH (r = -0.468, p <0.001), whereas significant positive correlation was seen with FSH (r = 0.332, p < 0.001).

Later, ROC analysis observed the discriminatory power of AMH along with FSH and AFC in two responder groups (Table-III). The AUROC for AMH was highest (0.771; *p* < 0.05) in terms of accurately discriminate between good and bad responders. FSH and AFC had a lower discriminatory power (0.692 and 0.690 respectively; *p <* 0.05). The AMH cut-off levels were calculated by the online software MedCalc^15^ for poor ovarian response. A value of 1.37ng/ml was calculated, with a specificity of 75% and a sensitivity of 90% to exhibit higher ability to discriminate between good and bad responders in all the groups. Serum AMH levels of less than 1.37ng/ml was associated with bad response while that of higher than 1.37ng/ml correlated better ovarian reserve.

## DISCUSSION

Young females are often advised to postpone infertility treatment based on their age and FSH levels. Hence, we explored strength of biomarkers including recently acclaimed AMH in discriminating between good and bad responder. Furthermore, we analyzed ovarian response in sub-groups segregated on the bases of age, FSH levels and AMH cut-off values calculated in our population.

In this study, we report significant correlation of oocytes retrieved with FSH, AMH and AFC while no association with age, BMI or LH. In comparison to FSH and AFC, AMH had a superior role as a solo marker of response. The AUROC for AMH was significantly higher than FSH and AFC (p <0.05). Our results revealed that 85.7% patients with normal AMH retrieved more than 5 oocytes. Furthermore, in unadjusted models of binary logistic regression, low AMH showed highest ability to identify bad responder as compared to raised FSH or AFC (6.66 times and 3.66 times respectively). Undoubtedly, AFC assessment is substantial to monitor infertility treatment however, operator’s variability is its limitation.^16^ As blood tests have marked advantages over ultrasound for primary care physicians, AMH has a greater efficiency over AFC, especially in setups where high class technology is not feasible.

While comparing AMH with the most commonly used marker FSH, segregation on the basis of FSH showed significant inverse correlation with AMH (Table-II). Therefore, we suggest AMH as its substitute in cases of deranged FSH production as discussed earlier. Being an “acyclic” marker, AMH assessment is considered reliable anytime throughout menstrual cycle^17^ although few studies do report converse findings.^18^

To further reinforce our results, AMH cut-off value for our population was calculated as 1.37ng/ml with the strength of 75% specificity and 90% sensitivity to correctly predict the response. We found that 87% patients having more than 1.37ng/ml AMH reported higher oocyte retrieval. Similarly, mean AMH of patients who conceived on ICSI was observed as 1.78 ± 0.95ng/ml. However, due to the limitation of sample size and inclusion of couples with male infertility, we cannot debate on wider role of AMH in predicting pregnancy outcomes. Various cut-off are reported across the globe. An Indian study has reported serum AMH levels of less than 1.4ng/ml as suggestive of poor ovarian response to stimulation.^19^ Here, it is important to emphasize that these cut-offs need to be used with caution as in our study we witnessed one patient reporting good response even with serum AMH of 0.6ng/ml. European study quoted AMH levels of as low as 0.8 µg/l as sufficient to reflect healthy ovarian response.^20^ This might be explained as although low AMH reflects decline in OR, but even few oocyte of good quality may still respond to gonadotropin stimulation. Thus, we advocate its capability in counselling infertile couples, selecting treatment protocol and tentatively predicting chances of pregnancy. 21

Next, we assessed AMH as a predictor of oocyte retrieval in two different groups based on age. In our study there was a stronger correlation in patients younger than 35 years (p<0.001, Fig.1 A and B). This further supports role of AMH to predict ART outcome in younger population that might be misjudged due to early age and a normal FSH. Studies do report that in population of similar age group, wide variations of OR have been testified in individuals.^3^ This explains the variation observed in AMH levels but its assessment along with other baseline investigations seems to be of additional value in screening young infertile patients with a decreased OR. Contrary to this, David H *et al*. reported significant correlation of AMH and oocyte retrieval in older infertile women.^22^ This difference could possibly be due to population stratification or selection criteria. Our study further reports similar results between FSH levels and oocyte retrieval as it was likewise found to correlate better in younger population (p<0.001, Fig.1 A and B). On the other hand, AFC showed higher correlation in the older group. Our results reported insignificant difference in AMH of patients with varied BMI, thus suggesting that it reflects true ovarian response irrespective of the patient’s BMI as also supported by earlier work.^23^

In conclusion, we support the role of AMH in reflecting the number of oocytes collected for successful ART. For the first time, we are reporting AMH levels in Pakistani infertile population and predict a cut-off value of 1.37ng/ml that discriminate good and bad responders. We strongly suggest incorporation of AMH evaluation in baseline assessment of ovarian reserve, especially in younger infertile patients as timely IVF treatment can improve the pregnancy outcomes in these populations. As our sample size was limited and localized, further longitudinal studies are required to reassess the cut-off values. Furthermore, studies focusing on association of serum AMH with viable pregnancy outcomes will increase the support for AMH as a predictive marker of live births in ART clinics.
